# Supplementary material for: Do Rare Genetic Conditions Exhibit a Specific Phonotype? A Comprehensive Description of the Vocal Traits Associated with Crisponi/Cold-Induced Sweating Syndrome Type 1
Source: Genes (Basel). 2025 Jul 26;16(8):881. doi: 10.3390/genes16080881 (PMC12385400; doi:10.3390/genes16080881)
Supplement: Supplementary file 1 [file genes-16-00881-s001.zip › genes-3748331-supplementary.pdf]

Supplementary Table S1. Demographic data for the main acoustical features extracted from the corner vowels /a/, /i/, and /u/ for both pathological and healthy groups, categorised by gender and age. The number of each subgroup is shown in brackets. Data highlighted in grey refer to statistically significant difference between the pathological subgroup and its related control. A (*) refers to a significant difference with p < .05, whereas (**) to a difference with p < .01.

/a/	F0 mean	Jitter	NNE	F1 mean	F2 mean	F3 mean
Crisponi - PS (7)	234±27	1.8±2.6	-21.0±6.0	926±206	1490±265	3871±200
Crisponi – AF (5)	206±19	3.4±3.3	-18.1±6.3	623±204	1297±85	2644±198
Crisponi - AM (1)	157	0.56	-18.3	781	1111	2725
PS – HS (20)	250±29	1.2±1.3	-21.7±3.5	1086±151	1440±108	3535±784
AF – HS (10)	205±22	1.6±2.2	-21.1±4.0	717±167	1249±212	2470±406
AM - HS (15)	119±29	0.5±1.2	-19.0±4.5	660±73	1089±67	2752±316
**/i/**	**F0 mean**	**Jitter**	**NNE**	**F1 mean**	**F2 mean**	**F3 mean**
Crisponi - PS (7)	257±25	2.9±3.6	-22.2±4.9*	549±59	2689±622	3829±140
Crisponi – AF (5)	208±19	2.2±3.2	-18.9±4.9**	376±41	2050±419	3051±374*
Crisponi - AM (1)	143	2.2	-21.8	321	1540	3211
PS – HS (20)	263±31	0.9±0.5	-27.2±4.9	563±62	2877±671	3748±233
AF – HS (10)	210±23	0.7±0.6	-26.3±4.2	343±39	2039±413	3287±422
AM – HS (15)	119±30	1.2±1.8	-25.6±2.7	318±21	1834±316	3085±379
**/u/**	**F0 mean**	**Jitter**	**NNE**	**F1 mean**	**F2 mean**	**F3 mean**
Crisponi - PS (7)	256±26	3.8±5.9	-23.3±4.6*	658±229	1372±150	3926±175
Crisponi – AF (5)	205±17	1.9±1.4	-20.0±4.5*	431±106	1234±160	2787±436
Crisponi – AM (1)	136	0.99	-24.8	459	917	3140
PS – HS (20)	262±30	1.0±1.0	-27.9±4.2	602±61	1374±198	3961±244
AF – HS (10)	210±25	1.1±2.0	-27.5±5.3	389±38	1165±223	3080±581
AM – HS (15)	121±33	0.6±0.3	-26.6±2.9	367±51	991±97	2889±318

PS = Paediatric Subjects, AF = Adult Females, AM = Adult Males, HS = Healthy Subjects, NNE= Normalized Noise Energy.
